# Robotic assisted minimally invasive esophagectomy versus minimally invasive esophagectomy

**DOI:** 10.3389/fonc.2023.1293645

**Published:** 2024-01-15

**Authors:** Mengchao Xue, Junjie Liu, Ming Lu, Huiying Zhang, Wen Liu, Hui Tian

**Affiliations:** Department of Thoracic Surgery, Qilu Hospital of Shandong University, Jinan, China

**Keywords:** da Vinci robot, robotic assisted minimally invasive esophagectomy, esophageal cancer, surgery, minimally invasive esophagectomy

## Abstract

**Background:**

Esophagectomy is the gold standard treatment for resectable esophageal cancer; however, there is insufficient evidence to indicate potential advantages over standard minimally invasive esophagectomy (MIE) in treating thoracic esophageal cancer. Robot-assisted minimally invasive esophagectomy (RAMIE) bridges the gap between open and minimally invasive surgery. In this single-center retrospective review, we compare the clinical outcomes of EC patients treated with MIE and RAMIE.

**Method:**

We retrospectively reviewed the clinical data of patients with esophageal cancer who underwent surgery at Qilu Hospital between August 2020 and August 2022, including 159 patients who underwent MIE and 35 patients who received RAMIE. The intraoperative, postoperative, and preoperative patient characteristics in both groups were evaluated.

**Results:**

Except for height, the MIE and RAMIE groups showed no significant differences in preoperative features (P>0.05). Further, there were no significant differences in intraoperative indices, including TNM stage of the resected tumor, tumor tissue type, or ASA score, between the two groups. However, statistically significant differences were found in some factors; the RAMIE group had a shorter operative time, less intraoperative bleeding, and more lymph nodes removed compared to the MIE group. Patients in the RAMIE group reported less discomfort and greater chest drainage on the first postoperative day than patients in the MIE group; however, there were no differences in other features between the two datasets.

**Conclusion:**

By comparing the clinical characteristics and outcomes of RAMIE with MIE, this study verified the feasibility and safety of RAMIE for esophageal cancer. Overall, RAMIE resulted in more complete lymph node clearance, shorter operating time, reduced surgical hemorrhage, reduced postoperative discomfort, and chest drainage alleviation in patients. To investigate the function of RAMIE in esophageal cancer, we propose undertaking a future clinical trial with long-term follow-up to analyze tumor clearance, recurrence, and survival after RAMIE.

## Introduction

Esophageal cancer (EC) is the sixth most common cause of cancer-related deaths worldwide, and the eighth most frequently diagnosed cancer (1, 2). China accounts for more than half of the global EC burden, with squamous cell carcinoma being the most common histological subtype (3). Esophageal cancer is highly malignant and has a poor prognosis. Currently, the majority of EC cases in China are middle- and late-stage ECs, which are treated with a comprehensive treatment strategy based on surgery with preoperative neoadjuvant therapy (such as chemotherapy, radiotherapy, and chemotherapy plus immunotherapy), minimally invasive or open-heart radical surgical resection plus lymph node dissection in the second field of the chest and abdomen or the third field of the neck, chest, and abdomen, followed by adjuvant chemotherapy, radiotherapy, radiotherapy, or immunotherapy depending on the postoperative pathological results (3).

The standard therapy for EC is surgical resection to achieve full primary tumor resection with radical lymph node dissection. In prior studies, the highest rates of full tumor eradication and long-term survival were achieved with transthoracic resection by esophagectomy (4, 5). However, esophagectomy is an invasive and difficult treatment that requires numerous entry points in the abdomen, chest, and neck, as well as significant technical surgical abilities. Furthermore, the invasive nature of treatments involving the chest and abdomen and the relatively high prevalence of postoperative complications are significant issues with esophagectomy (5, 6). The use of thoracoscopy or laparoscopy in minimally invasive esophagectomy (MIE) reduces surgical damage to the chest or abdominal wall (7). Indeed, several randomized controlled studies and meta-analyses have shown that MIE lowers postoperative complications, especially pulmonary problems, and has long-term survival results equivalent to open esophagectomy (8–10). However, MIE is a technically difficult treatment that can only be performed by highly competent surgeons.

Transthoracic robot-assisted minimally invasive esophagectomy (RAMIE) has recently arisen as an additional surgical option for MIE due to new advances in robotics (11, 12). Robotic procedures have various technological benefits over thoracoscopic procedures, including increased surgical accuracy and mobility in the limited environment of the mediastinum allowed by the flexibility of the four arms used in RAMIE. Further, superior image quality, including three-dimensional vision, can be achieved. Because of these benefits, RAMIE is regarded as the best technique for aggressive tumor surgery (12–16). However, the benefits of RAMIE over minimally invasive esophagectomy (MIE) have not been well characterized; as a result, RAMIE has not been extensively employed in the treatment of EC. Therefore, the study aimed to evaluate the results of RAMIE and MIE and to identify any clinical or oncological advantage of RAMIE against MIE in EC, as well as to investigate the feasibility and safety of MIE using the da Vinci robotic system in EC patients.

## Patients and methods

Prior to surgery, all patients completed an informed consent form for the use of their clinical information, which was authorized by the Ethics Committee of Qilu Hospital, Shandong University (registration number: KYLL-202008-023-1).

### Patient selection

This study reviewed a prospectively collected database of patients with esophageal cancer who underwent surgery at Qilu Hospital between August 2020 and August 2022. The inclusion criteria for this study were: (1) EC confirmed by preoperative imaging and histopathological findings, (2) treatment with MIE or RAMIE, and (3) complete available clinical and pathological data. The exclusion criteria were (1) age ≥80 years, (2) presence of distant metastases, and (3) presence of significant pulmonary comorbidities.

### Data collection and variable definitions

The following data was collected from eligible patients registered in the Qilu Hospital database: (1) General clinical data: age, sex, preoperative comorbidities (hypertension, diabetes, chronic obstructive pulmonary disease [COPD]), history of smoking, history of alcohol consumption, ASA score, and body mass index (BMI). (2) Preoperative assessment data, including the presence or absence of preoperative symptoms, tumor location, and PS score. (3) Intraoperative related data: operative time and intraoperative bleeding volume. (4) Postoperative assessment data: pain score on the first postoperative day, gastric and chest tube drainage flow on the third postoperative day, transfer to the ICU, removal of chest and gastric tubes on the first postoperative day, length of hospital stay, postoperative drinking and eating time, and postoperative complications. (5) Postoperative pathological results: TNM stage, tumor histological type, number of cleared lymph nodes, and number of positive results.

All minimally invasive portions of the surgical procedures were performed by the same experienced laparoscopic surgeon with two surgical assistants. General anesthesia was combined with thoracic epidural anesthesia to minimize pain intra-and post-operatively.

### Surgical procedure for MIE

(1) First, the patient’s esophagus was freed with thoracoscopic assistance, and mediastinal lymph node dissection was performed.

**Figure 1:**
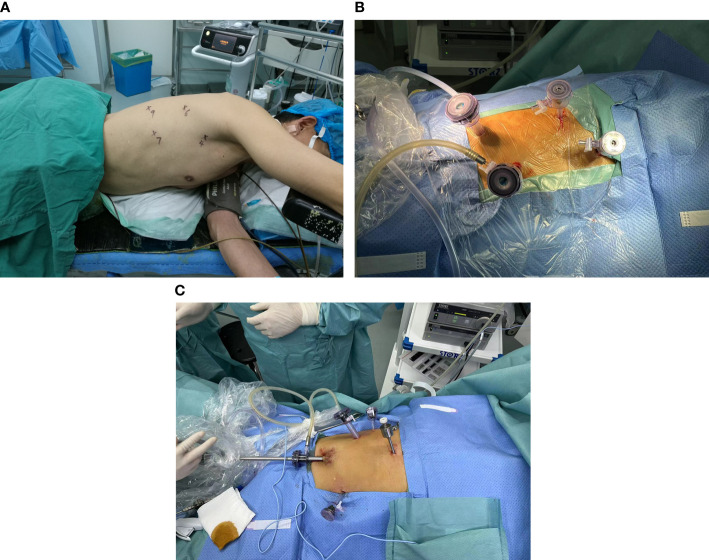
**(A)** Schematic diagram of the chest incision for MIE. **(B)** Picture of chest incision during surgery. **(C)** Schematic diagram of the abdominal incision for MIE.

After tracheal intubation, the patient was adjusted to the left lateral recumbent position with a slight forward tilt to facilitate exposure of the esophageal bed, and an axillary cushion placed under the patient’s armpit to facilitate abdominal breathing. Four thoracoscopic incisions were made. A 1.5-cm incision was made in the patient’s seventh intercostal space in the axillary midline, through which a 12-mm trocar was placed as an observation hole, while a the thoracoscopic optic was inserted into the observation hole to observe the thoracic cavity for adhesions. Subsequently, a 1.5-cm incision was made in the patient’s ninth intercostal space along the scapular line, and a 12-mm trocar was placed as an auxiliary operative hole. A 5-mm trocar was placed in the fourth intercostal space in the axillary midline as the first operative hole. Another 5-mm trocar was placed between the sixth intercostal space in the scapular line as the second operative site. The locations of the above four incisions are shown in **Figure 1A, B**. The electrocoagulation hook, ultrasonic knife, and other instruments were accessed to separate the adhesions and free the esophagus. The assistant applied an auxiliary manipulation card to assist in freeing the esophagus by pressing the right lung anteriorly to fully expose the esophageal bed using a trephine clamp. During freeing of the esophagus, the azygos vein was bluntly freed to separate it from the surrounding tissues and closed using a cutting occluder. The scope of surgical resection included the thoracic esophagus and esophageal tumor, paraesophageal lymph nodes, adipose tissue of the esophageal bed, and subcarinal nodes in their entirety. After resection, the para-recurrent laryngeal nerve nodes were further cleared, and the cleared lymph nodes were removed and placed in a specimen bag, and the rubber gloves were replaced. After adequate rinsing with saline and electrocoagulation, the hemorrhage stopped. The incision was closed by ventilating both lungs, placing a chest drain in the observation hole, and suturing the incision with an external Waterseal bottle.

(2) Next, the stomach was freed, and the associated lymph nodes were cleared under laparoscopic assistance.

After completion of the chest maneuver, the patient position was changed from supine to the reverse Trendelenburg position. The patient’s head was tilted slightly to the right to allow for subsequent neck manipulation and to maintain a small tidal volume of ventilation in both lungs. An incision was made at the patient’s lower umbilical margin, a CO_2_ pneumoperitoneum of 8 mmHg was created while a 12-mm trocar was simultaneously placed and a 30° optic was placed in this hole as an observation hole. Two cm above the level of the umbilicus in the midclavicular line on the left side of the patient and 2 cm below the rib margin, 12- and 5-mm trocars were placed as the first and second operative holes, respectively. Further, a 5-mm trocar was placed 2 cm below the rib margin in the right midclavicular line as an auxiliary surgical hole. A 5-mm trocar was also placed subxiphoidally as an auxiliary surgical hole. The locations of the above five incisions are shown in **Figure 1C**. Subsequently, a subxiphoid hepatic round ligament suspension was performed, and the lesser curvature of the stomach was exposed. The greater and lesser curvatures of the stomach were freed, the associated lymph nodes were cleared, the left gastric vessel was exposed, and the left gastric artery was closed using a cutting closure. The esophagus was then pulled down through the esophageal hiatus, and the patient’s subxiphoid incision was extended to 5 cm, thus pulling the patient’s stomach out of the body. Subsequently, the length of the preserved stomach was determined, and a tubular stomach with a width of 3 cm was created using linear cutting closure.

(3) Finally, a routine neck anastomosis was performed.

### Surgical procedure for RAMIE

(1) First, the patient’s thoracic segmental esophagus was freed using a da Vinci robot and mediastinal lymph node dissection was performed.

After completion of tracheal intubation, the patient was placed in the left lateral position; if this did not ensure sufficient exposure of the esophagus, an anteriorly tilted position was applied. A 10-mm incision was made in the patient’s mid-axillary line between the 5th intercostal ribs to allow placement of a da Vinci robotic lumenscope, and thoracic exploration was performed to determine the presence or absence of thoracic adhesions. Subsequently, two 8-mm incisions were made in the anterior axillary line at the 3rd intercostal space and in the posterior axillary line at the 7th intercostal space, and sheath cards were placed on the right and left arms, respectively. The esophagus was then separated by placing an ultrasonic knife on the right arm and grasping forceps on the left arm. At the same time, a 10-mm incision was made in the anterior axillary line at the 7th intercostal space, which served as an assistant’s hole through which the assistant placed luminal grasping forceps to pull the right lung anteriorly to further expose the esophageal bed and assist in freeing the esophagus. Dissection of the mediastinal pleura was performed based on the patient’s esophageal alignment. After a thorough examination of the tumor to ensure that there was no obvious extravasation, the esophagus was detached at the lower end of the normal esophagus. After separation, robotic grasping forceps were applied in conjunction with an ultrasonic knife to free the arch of the azygos vein to separate it from the surrounding tissue, and the azygos vein was cut using a cutting occluder. The esophagus was fully freed upward to the top of the chest and downward to the diaphragmatic hiatus, and the diaphragmatic hiatus was fully opened to facilitate esophageal pull-down during laparotomy. After completion of esophageal freeing, the thoracic lymph nodes, including the bilateral para-recurrent laryngeal nerve nodes, paraesophageal nodes, and subcarinal nodes, were cleared, and complete resection was performed. The cleared lymph nodes were removed and placed in a specimen bag, and the rubber gloves were replaced. The patient was examined, and after adequate saline rinsing, electrocoagulation was performed to halt the bleeding. Bipulmonary ventilation was performed, a chest drain was placed in the caval orifice, an external water-sealed bottle was attached, and the remainder of the incision was sutured.

(2) Second, the stomach was freed.

After completion of the chest maneuver, small tidal volume ventilation of both lungs was performed in a flat position, with the patient in the reverse Trendelenburg position, with the head tilted slightly to the right side to facilitate the neck maneuver. Incisions were made 1 cm below the patient’s umbilicus on the left side, 2 cm below the rib arch in the left anterior axillary line, flat umbilicus in the left midclavicular line, flat umbilicus in the right midclavicular line, and below the rib arch in the right anterior axillary line. These incisions were used as the entrance to the luminal scotomical sheath card for the da Vinci robot, right robotic arm scotomical incision, assistant scotomical incision, left robotic arm scotomical incision, and third robotic arm scotomical incision, respectively. An artificial pneumoperitoneum was established, with pressure maintained at 10–12 cm H_2_O. The greater and lesser curvatures of the stomach were freed using an ultrasonic knife, the right vascular arch of the gastric omentum was preserved, the associated lymph nodes were cleared, the left gastric vessels were exposed, and the left gastric artery was cut and closed with a cutting occluder. The lower esophagus was then adequately freed from the diaphragm. The patient’s abdominal cavity was checked for bleeding, adequate hemostasis was achieved using an ultrasonic scalpel, and operation of the da Vinci robotic system was completed. A longitudinal incision was made 5 cm below the patient’s xiphoid process, the patient’s stomach was pulled out of the body, the length of the stomach to be preserved was determined, and the stomach was cut 3 cm below the cardia. Creation of a tubular stomach was initiated using a linear cutting obturator with a width of 3 cm. Before the cervical esophagectomy, we set a pull line and sutured the finished tube stomach to the pull line. We applied a pull line to the neck incision and pulled the tube stomach to the neck. We pulled the tubular stomach out of the mediastinum through the esophageal hiatus. A triple-stitched marking suture was made at the highest point of the fundus of the stomach to tie it to the suture line of the esophageal stump.

(3) Finally, routine neck anastomosis was performed.

### Neck anastomosis surgical procedure

Currently, most neck anastomoses, including those performed in our hospital, use the circular stapling technique (CS) for tubular anastomoses.

CS was performed as follows: The broken end of the esophagus was clamped with a bag clamp, and the broken end of the esophagus was sutured with a bag line. The stapler thimble was then placed at the cut end of the esophagus and fixed with a purse-string suture. A 2-cm incision was made at the top of the stomach tube. When the stapler was inserted into the stomach tube, the proper position of the greater curvature side of the stomach was selected, and mechanical anastomosis was performed with the stapler thimble. An incision was made at the top of the stomach and closed.

### Statistical analysis

The data for this study were recorded and described using Microsoft Excel (Microsoft Corporation, Redmond, WA, USA). All statistical analyses were performed using SPSS 26.0 (SPSS Inc., Chicago, Illinois, USA) and R statistical software (Windows version 4.2.1, http://www.r-project.org/). Normally distributed continuous variables are expressed as the mean ± standard deviation (SD) and compared using the Student’s t-test. For non-normally distributed continuous variables, data are expressed as the medians (interquartile range [IQR]) and were compared between the two groups using the Mann–Whitney U test. Categorical variables were compared using Pearson’s chi-square test or Fisher’s exact test. Statistical significance was defined as a two-sided P-value of less than 0.05.

## Results

This study included 159 patients who underwent minimally invasive esophagectomy and 35 who underwent robot-assisted minimally invasive esophagectomy. The choice of surgical approach was made based on the preferences of both the surgeon and the patient. The results of this study are preliminary and inconclusive. Future investigations are necessary to validate the kinds of conclusions that can be drawn from this study.

### Preoperative characteristics

**Table 1** shows the preoperative characteristics of the two patient groups. As shown in **Table 1**, EC was significantly more common in males than females. The percentages of smokers and alcoholic drinkers were high in both treatment groups. Regarding initial symptoms, feelings of obstruction in eating and dysphagia were the most common, while gastrointestinal symptoms such as acid reflux, belching, and abdominal distension were less common, and a small number of patients also displayed symptoms of laryngeal nerve damage, such as choking and coughing with drinking water and hoarseness. Tumors were most commonly located in the lower thoracic esophagus, followed by the middle thoracic esophagus, while the upper thoracic esophagus was the least common. Preoperative neoadjuvant treatment was administrated in 19.1% of the patients. A total of 91.2% of patients were classified as having an ASA score of II or above. The median age of the patients was 64 years, and the median BMI was 23.43 kg/m^2^. The mean PS score was 80. The median distances from the central incisors were 30.5 cm. Except for height, which might be attributed to identical preoperative features in the two groups, all P values between the MIE and RAMIE groups were >0.05.

**Table 1:** Comparison of each preoperative characteristic between the two groups of patients.

Variables	Overall (N=194)	MIE (N=159)	RAMIE (N=35)	p
Gender, n (%)				0.636
Female	44 (22.7)	35 (22.0)	9 (25.7)	
Male	150 (77.3)	124 (78.0)	26 (74.3)	
Smoking history, n (%)				0.344
Non-smoker	75 (38.7)	59 (37.1)	16 (45.7)	
Smoker	119 (61.3)	100 (62.9)	19 (54.3)	
Drinking history, n (%)				0.236
Non-Drinker	77 (39.7)	60 (37.7)	17 (48.6)	
Drinker	117 (60.3)	99 (62.3)	18 (51.4)	
Hypertension, n (%)				0.056
No	141 (72.7)	111 (69.8)	30 (85.7)	
Yes	53 (27.3)	48 (30.2)	5 (14.3)	
Diabetes, n (%)				0.367
No	182 (93.8)	148 (93.1)	34 (97.1)	
Yes	12 (6.2)	11 (6.9)	1 (2.9)	
Coronary heart disease, n (%)				0.367
No	182 (93.8)	148 (93.1)	34 (97.1)	
Yes	12 (6.2)	11 (6.9)	1 (2.9)	
Other preoperative comorbidities, n (%)				0.323
No	175 (90.2)	145 (91.2)	30 (85.7)	
Yes	19 (9.8)	14 (8.8)	5 (14.3)	
Feeling of obstruction in eating/Difficulty in eating, n (%)				0.599
No	57 (29.4)	48 (30.2)	9 (25.7)	
Yes	137 (70.6)	111 (69.8)	26 (74.3)	
Acid reflux belching, n (%)				0.939
No	178 (91.8)	146 (91.8)	32 (91.4)	
Yes	16 (8.2)	13 (8.2)	3 (8.6)	
Abdominal pain/Eating pain/Rear sternal pain, n (%)				0.814
No	141 (72.7)	115 (72.3)	26 (74.3)	
Yes	53 (27.3)	44 (27.7)	9 (25.7)	
Bloating, n (%)				0.128
No	184 (94.8)	149 (93.7)	35 (100.0)	
Yes	10 (5.2)	10 (6.3)	0 (0.0)	
Choking and coughing with wate/Hoarseness, n (%)				0.243
No	188 (96.9)	153 (96.2)	35 (100.0)	
Yes	6 (3.1)	6 (3.8)	0 (0.0)	
Tumor site, n (%)				0.926
Upper thoracic	19 (9.8)	15 (9.4)	4 (11.4)	
Mid thoracic	60 (30.9)	49 (30.8)	11 (31.4)	
Lower thoracic	115 (59.3)	95 (59.7)	20 (57.1)	
Preoperative neoadjuvant therapy, n (%)				0.204
No	157 (80.9)	126 (79.2)	31 (88.6)	
Yes	37 (19.1)	33 (20.8)	4 (11.4)	
ASA Score, n (%)				0.129
I	3 (1.5)	3 (1.9)	0 (0.0)	
II	177 (91.2)	142 (89.3)	35 (100.0)	
III	14 (7.2)	14 (8.8)	0 (0.0)	
Age (years), median (IQR)	64.00 (58.00, 69.00)	64.00 (58.00, 69.00)	64.00 (56.50, 69.50)	0.923
weight (kg), median (IQR)	65.00 (59.00, 73.75)	65.00 (59.00, 75.00)	64.00 (58.50, 70.00)	0.433
Height (m), median (IQR)	1.68 (1.60, 1.72)	1.70 (1.62, 1.72)	1.65 (1.60, 1.69)	0.038
BMI (kg/m2), median (IQR)	23.43 (21.46, 25.67)	23.45 (21.47, 25.66)	23.23 (21.50, 25.55)	0.854
Distance from the median incisor (cm), median (IQR)	30.50 (27.50, 33.00)	30.50 (27.50, 33.00)	30.00 (27.50, 33.00)	0.728
PS Rating, median (IQR)	80.00 (80.00, 90.00)	80.00 (80.00, 90.00)	80.00 (80.00, 90.00)	0.683

ASA, American Society of Anesthesiologists; BMI, body mass index; PS rating, performance status rating; MIE, minimally invasive esophagectomy; RAMIE, robot-assisted minimally invasive esophagectomy.

### Intraoperative index comparison

**Figure 2:**
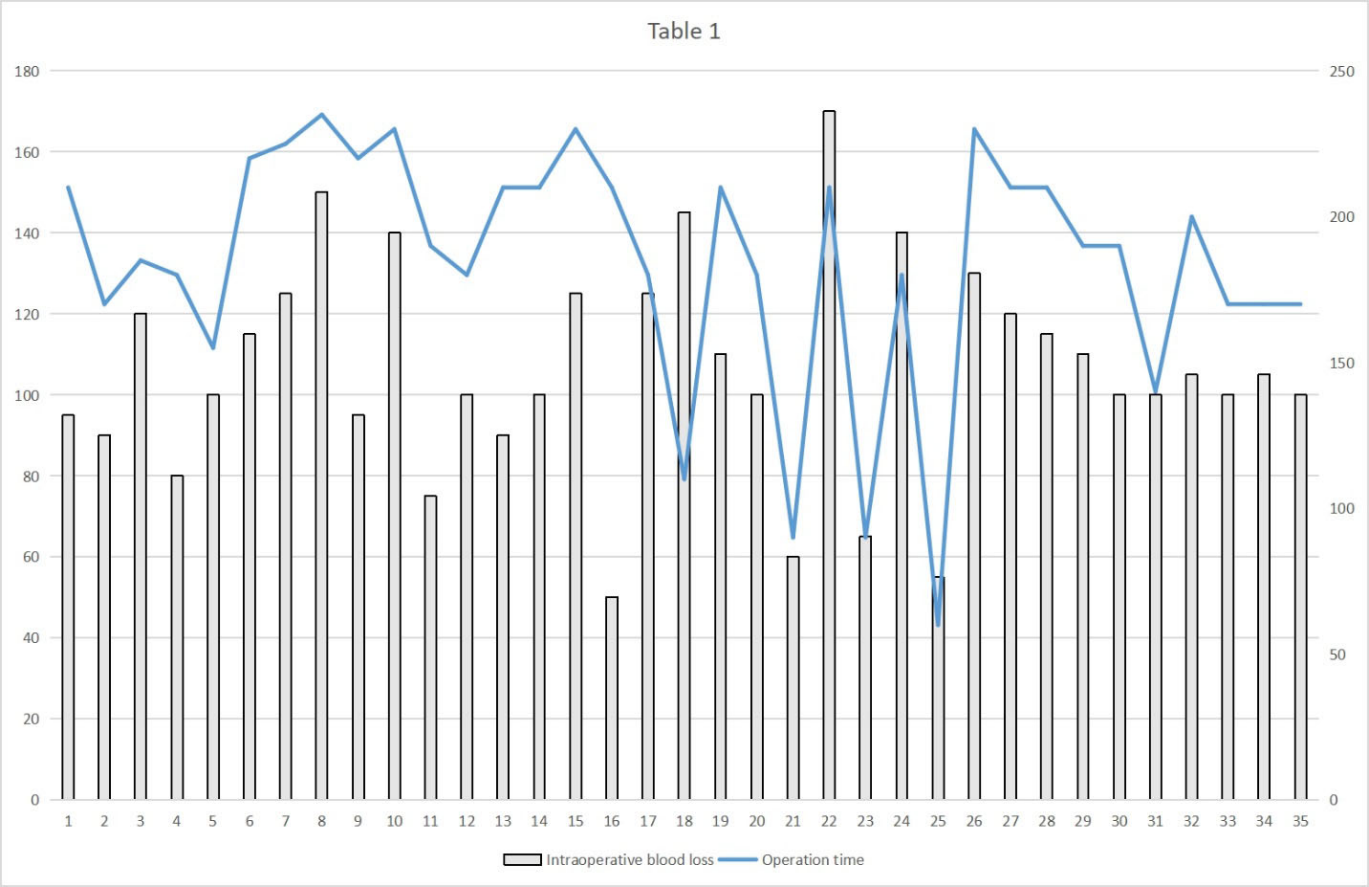
The operative time and blood loss in the RAMIE group.

**Table 2** provides detailed information on the comparisons between the two treatment groups. There were no significant differences between the two groups in terms of TNM stage of the resected tumor, tumor tissue type, or ASA score. However, in terms of operating time, intraoperative hemorrhage, total number of lymph nodes removed, and the number of lymph nodes removed from the chest and belly, statistically significant differences were found between the MIE and RAMIE groups. The mean surgical duration in the MIE group was 210 min, whereas the RAMIE group required considerably less time (p=0.007) at 190 min. In terms of intraoperative bleeding, the MIE group had a mean bleeding volume of 120 mL compared with 100 mL in the RAMIE group (p = 0.006). The mean number of lymph nodes removed in the MIE group was 19, compared to 23 in the RAMIE group (p = 0.001). The RAMIE group also exhibited a considerable increase in the number of lymph nodes removed from the chest and abdomen. However, the two groups showed comparable results in terms of the number of positive lymph nodes excised, with no statistically significant differences. **Figure 2** shows the operative time and blood loss in all 35 patients in the RAMIE group.

**Table 2:** Comparison of various intraoperative characteristics between the two groups of patients.

Variables	Overall (N=194)	MIE (N=159)	RAMIE (N=35)	p
Pathologic T stages (%)				0.817
Tis	21 (10.8)	19 (11.9)	2 (5.7)	
T1a	32 (16.5)	25 (15.7)	7 (20.0)	
T1b	23 (11.9)	19 (11.9)	4 (11.4)	
T2	42 (21.6)	35 (22.0)	7 (20.0)	
T3	76 (39.2)	61 (38.4)	15 (42.9)	
Pathologic N stages (%)				0.888
N0	121 (62.4)	99 (62.3)	22 (62.9)	
N1	44 (22.7)	37 (23.3)	7 (20.0)	
N2	26 (13.4)	21 (13.2)	5 (14.3)	
N3	3 (1.5)	2 (1.3)	1 (2.9)	
Pathologic M stages (%)				NA
M0	194 (100.0)	159 (100.0)	35 (100.0)	
M1	0(0.0)	0(0.0)	0(0.0)	
Pathologic data (%)				0.69
Tis	21 (10.8)	19 (11.9)	2 (5.7)	
IA	10 (5.2)	8 (5.0)	2 (5.7)	
IB	37 (19.1)	31 (19.5)	6 (17.1)	
IC	1 (0.5)	1 (0.6)	0 (0.0)	
IIA	43 (22.2)	31 (19.5)	12 (34.3)	
IIB	17 (8.8)	15 (9.4)	2 (5.7)	
IIIA	14 (7.2)	11 (6.9)	3 (8.6)	
IIIB	48 (24.7)	41 (25.8)	7 (20.0)	
IVA	3 (1.5)	2 (1.3)	1 (2.9)	
Tumor tissue type (%)				0.461
Squamous carcinoma	187 (96.4)	154 (96.9)	33 (94.3)	
Adenocarcinoma	7 (3.6)	5 (3.1)	2 (5.7)	
ASA Score (%)				0.129
1	3 (1.5)	3 (1.9)	0 (0.0)	
2	177 (91.2)	142 (89.3)	35 (100.0)	
3	14 (7.2)	14 (8.8)	0 (0.0)	
Operation time (min, median (IQR))	210.00 (180.00, 220.00)	210.00 (180.00, 227.50)	190.00 (170.00, 210.00)	0.007
Intraoperative blood loss (mL, median (IQR))	120.00 (100.00, 148.75)	120.00 (100.00, 150.00)	100.00 (95.00, 122.50)	0.006
Total number of lymph nodes removed (median (IQR))	20.00 (16.00, 25.00)	19.00 (15.00, 24.00)	23.00 (22.00, 26.50)	<0.001
Total number of chest lymph nodes removed (median (IQR))	12.00 (9.00, 15.00)	11.00 (9.00, 14.50)	14.00 (12.00, 18.00)	<0.001
Total number of abdominal lymph nodes removed (median (IQR))	8.00 (6.00, 10.75)	8.00 (5.00, 10.00)	10.00 (8.00, 11.00)	0.008
Total number of positive lymph nodes excised (median (IQR))	0.00 (0.00, 1.00)	0.00 (0.00, 1.00)	0.00 (0.00, 2.00)	0.871
Total number of positive lymph nodes removed from the chest (median (IQR))	0.00 (0.00, 1.00)	0.00 (0.00, 1.00)	0.00 (0.00, 1.00)	0.997
Total number of positive abdominal lymph nodes excised (median (IQR))	0.00 (0.00, 0.00)	0.00 (0.00, 0.00)	0.00 (0.00, 0.00)	0.459

MIE, minimally invasive esophagectomy; RAMIE, robotic-assisted minimally invasive esophagectomy.

### Comparison of postoperative indicators

**Table 3** compares the postoperative characteristics of the two groups. Our research found statistically significant differences in pain levels and chest drainage on the first postoperative day, but no changes in any other features between the two data groups. Patients in the RAMIE group reported lower pain levels and required a higher number of chest drains on the first postoperative day than those in the MIE group. There were no statistically significant differences in the postoperative hospital stay, postoperative feeding time, or postoperative chest drain removal time between the groups. Further, no cases in either group required postoperative transfer to the intensive care unit, and none of the patients experienced celiac disease or laryngeal nerve damage. The risk group had a greater risk of anastomotic fistula, while the MIE group had a higher rate of respiratory problems. However, no statistically significant differences in postoperative complications were observed between the groups.

**Table 3:** Comparison of various postoperative characteristics between the two groups of patients.

Variables	Overall (N=194)	MIE (N=159)	RAMIE (N=35)	p
Pain score on the first postoperative day (day, median (IQR))	6.00 (6.00, 6.00)	6.00 (6.00, 6.00)	6.00 (5.00, 6.00)	<0.001
Chest tube drainage on the first postoperative day (day, median (IQR))	300.00 (200.00, 400.00)	260.00 (200.00, 400.00)	360.00 (300.00, 430.00)	0.004
Gastric tube drainage on the first postoperative day (day, median (IQR))	25.00 (15.00, 50.00)	25.00 (15.00, 50.00)	20.00 (20.00, 67.50)	0.463
Chest tube drainage on the second postoperative day (day, median (IQR))	200.00 (132.50, 297.50)	200.00 (135.00, 300.00)	200.00 (135.00, 260.00)	0.822
Gastric tube drainage on the second postoperative day (day, median (IQR))	52.50 (30.00, 120.00)	60.00 (30.00, 120.00)	50.00 (25.00, 100.00)	0.292
Chest tube drainage on the third postoperative day (day, median (IQR))	140.00 (100.00, 200.00)	140.00 (100.00, 200.00)	120.00 (100.00, 200.00)	0.993
Gastric tube drainage on the third postoperative day (day, median (IQR))	60.00 (30.00, 130.00)	65.00 (30.00, 140.00)	50.00 (17.50, 100.00)	0.205
Postoperative chest tube removal time (day, median (IQR))	10.00 (8.00, 12.00)	10.00 (8.00, 12.00)	10.00 (9.00, 12.00)	0.55
Postoperative gastric tube removal time (day, median (IQR))	7.00 (6.00, 8.00)	7.00 (6.00, 8.00)	7.00 (6.00, 8.00)	0.667
Postoperative feeding time (day, median (IQR))	8.00 (7.00, 10.00)	8.00 (7.00, 10.00)	8.00 (7.00, 10.50)	0.405
Postoperative hospitalization time (day, median (IQR))	11.00 (10.00, 13.00)	11.00 (10.00, 13.00)	11.00 (10.00, 13.00)	0.766
Postoperative complications (%)				0.336
Absence	154 (79.4)	126 (79.2)	28 (80.0)	
Anastomotic fistula	18 (9.3)	12 (7.5)	6 (17.1)	
Celiac disease	0 (0.0)	0 (0.0)	0 (0.0)	
Recurrent laryngeal nerve injury	0 (0.0)	0 (0.0)	0 (0.0)	
Respiratory complications	15 (7.7)	14 (8.8)	1 (2.9)	
Digestive system complications	1 (0.5)	1 (0.6)	0 (0.0)	
Incisional complications	3 (1.5)	3 (1.9)	0 (0.0)	
Others	3 (1.5)	3 (1.9)	0 (0.0)	
Whether to transfer to ICU (%)				NA
No	194 (100.0)	159 (100.0)	35 (100.0)	
Yes	0 (0.0)	0 (0.0)	0 (0.0)	

MIE, minimally invasive esophagectomy; RAMIE, robotic-assisted minimally invasive esophagectomy.

## Discussion

EC is seventh most common malignancy, with the sixth highest fatality rate worldwide (17). In China, males are more likely than women to develop EC, and its prevalence is greater in rural than in metropolitan regions (18). The two most common pathological types of esophageal cancer are squamous carcinoma and adenocarcinoma. In Asian countries with a high incidence of EC, such as China, squamous carcinoma accounts for more than 90% of cases; conversely, adenocarcinoma is the most common pathological form in Western countries (19). As a very aggressive malignant tumor, esophageal cancer is often associated with widespread lymph node metastases, resulting in poor prognosis (20, 21). Therefore, extensive esophageal cancer resection and sufficient lymph node dissection reinforced by preoperative or postoperative radiation are the most common therapeutic options.

Surgery is still the primary therapeutic option for patients with resectable EC (8, 22–24). Various surgical techniques can be used to perform radical esophageal cancer surgery (25–27). Traditional open surgery is stressful and has a high incidence of postoperative complications, morbidity, and death, all of which exert a significant negative impact on patients’ quality of life (28–30). By opening the chest and belly, thoracolaparoscopic radical esophageal cancer surgery reduces the enormous stress on patients and enhances their recovery (30–32). However, this surgical technique is also limited by the two-dimensional field of view, requirement for long and straight instruments, arm tremors, and other shortcomings of the laparoscope, making it extremely difficult for primary surgeons to operate in narrow spaces, such as clearing the upper mediastinal lymph nodes. Further, this technique is associated with a long learning curve and a high incidence of complications such as laryngeal nerve injury (33–36). Consequently, several medical institutes have developed and generally adopted da Vinci robot-assisted minimally invasive esophageal cancer resection (37–39).

The da Vinci Surgical Robot System is one of the most complicated and expensive surgical systems available in the market. This system comprises an integrated surgeon’s workstation, a bedside robotic arm system, and a high-definition imaging system. Robotic surgery is highly sophisticated, involving the combination of cutting-edge technology with clinical surgery (40, 41), and is widely used in the field of surgical oncology treatment (42–44). In 2003, the da Vinci robot was first applied to esophageal cancer resection (45); thereafter, the results of several studies, both domestic and international, showed that the da Vinci robot-assisted minimally invasive esophageal cancer resection is safe and feasible. Further, this system has been associated with several significant advantages, including reduced intraoperative bleeding, better protection of the laryngeal recurrent nerve, and more thorough lymph node clearance compared to ordinary MIE (16, 39, 46–49). Following these results, an international consensus on the utility of RAMIE resection was formed (50, 51).

Research on robot-assisted minimally invasive esophagectomy has progressed rapidly since the first robot-assisted minimally invasive esophagectomy was performed by Van Hillegersberg et al. (52). In one study, Kim et al. reported the results of RAMIE performed by oncologic surgeons in the prone position on 21 patients with esophageal cancer, finding that robotic-assisted minimally invasive esophagectomy had shorter operative times, less bleeding, and no pulmonary complications, and the number of lymph nodes dissected was 38.0 ± 14.2 (13). Puntambekar et al. performed the RAMIE procedure in 32 patients and reported satisfactory results, as follows: mean operative time, 210 min; mean total bleeding, 80 mL; postoperative pulmonary complications, 6.2%; and postoperative hospitalization, 9 days (14). Suda et al. further conducted a prospective study of 36 patients with squamous esophageal cancer who underwent robot-assisted treatment with thoracoscopic radical esophagectomy, finding a significantly lower frequency of recurrent nerve palsy after robot-assisted surgery than after normal minimally invasive esophagectomy (53). This is due to the enhanced surgical ergonomics and anatomy as a result of 3D imaging of the robotic arm and operative region. In another study, Cerfolio et al. performed robotic Ivor Lewis esophagectomy with robotic-assisted 2-layer hand-sewn esophagogastric anastomosis in 22 patients, concluding that robotic thoracic esophagectomy using more than just ports is feasible and safe, allowing for complete R0 dissection with thoracic lymph node dissection and enabling early suturing of the 2-layer thoracic anastomosis (54). In 2021, Manigrasso et al. conducted a systematic evaluation and meta-analysis of robotic esophagectomy, and concluded that robotic surgery was superior to open and conventional laparoscopic surgery because of the fewer postoperative complications, better oncologic outcomes, less postoperative pneumonia, and greater number of harvested lymph nodes (16). However, many studies in this area of anastomotic leakage (AL) remain highly controversial. Several studies have suggested that RAMIE may increase the incidence of postoperative AL compared to MIE or open surgery (33).

Our data indicates that RAMIE is a viable and safe treatment for patients with esophageal cancer. Comparison of intraoperative indicators revealed that patients who underwent RAMIE had a shorter surgical procedure time (average, 190 min) and less intraoperative hemorrhage (average, 100 ml). Conversely, RAMIE increased the operational time by 160 min in Yoshiaki Osaka’s trial; however, the overall blood loss and bleeding from thoracic manipulation greatly decreased (55). The increase in robot-assisted surgery time is attributed to the extra time required for preparation, such as rolling in and docking the da Vinci system and performing the surgery while manipulating the microvasculature to confirm the separation layer with the magnified visualization of the da Vinci system. In contrast, because the surgeon in our institute is highly skilled and experienced with this platform, our robot-assisted surgery required very little time for the machine and instrumentation setup, and the complexity of the process considerably decreased with the aid of the robot, resulting in a reduction in procedure time. Our study also found that robot-assisted esophagectomy resulted in the removal of an average of 23 lymph nodes, including those in the chest and belly. Second, when postoperative parameters were compared, robot-assisted esophagectomy performed better. For example, patients in the RAMIE group showed fewer pain ratings with a mean score of 6 on the first postoperative day. This is probably due to the fact that RAMIE is less invasive for the patients, resulting in less postoperative pain. However, several elements were inferior to those of the MIE. On the first postoperative day, we noted increased chest drainage, with an average volume of 360 mL. This may be due to the more complete lymph node dissection. Moreover, our study did not achieve statistically significant outcomes in terms of postoperative length of stay and postoperative complications, which were probably due to the limited sample size.

This study had some limitations. First, the research group was diverse, with only a limited number of patients in the cohort. However, while it is true that the number of RAMIE groups included in this study is small, considering the small number of cases accumulated over the years, this cohort is nevertheless valuable. The second drawback derives from the retrospective study design; although the preoperative parameters in both groups were equivalent, we cannot exclude the possibility of unexplained, uncontrolled selection bias. Third, this was a non-randomized comparative study with a significant bias in the surgical technique selection for RAMIE or MIE. Finally, we did not follow-up the patients sufficiently to investigate the postoperative recurrence rates and survival in the short and long terms.

## Conclusion

This prospective pilot study compared the clinical outcomes of RAMIE vs MIE for EC to validate the feasibility and safety of RAMIE as a valid treatment for EC. Our findings indicate that RAMIE resulted in more comprehensive lymph node dissection, shorter operating time, reduced surgical hemorrhage, and alleviation of postoperative pain and chest drainage in patients compared to MIE. Although striking a balance between radicalism and safety is an essential objective of minimally invasive surgery, postoperative follow-up findings were not assessed in the present study. To investigate the function of RAMIE in esophageal cancer, we propose undertaking the next clinical trial with long-term follow-up to analyze tumor clearance, recurrence, and survival rates after receiving RAMIE.

## Data availability statement

The original contributions presented in the study are included in the article/supplementary material. Further inquiries can be directed to the corresponding author.

## Ethics statement

The studies involving humans were approved by the Ethics Committee of Qilu Hospital, Shandong University (registration number: KYLL-202008-023-1). The studies were conducted in accordance with the local legislation and institutional requirements. Written informed consent for participation was not required from the participants or the participants’ legal guardians/next of kin in accordance with the national legislation and institutional requirements.

## Author contributions

MX: Conceptualization, Data curation, Formal analysis, Investigation, Methodology, Project administration, Resources, Software, Supervision, Validation, Visualization, Writing – original draft, Writing – review & editing. JL: Conceptualization, Data curation, Investigation, Methodology, Project administration, Writing – review & editing. ML: Formal analysis, Resources, Software, Supervision, Validation, Visualization, Writing – original draft, Writing – review & editing. HZ: Conceptualization, Methodology, Validation, Visualization, Writing – review & editing. WL: Conceptualization, Investigation, Methodology, Project administration, Resources, Software, Supervision, Validation, Visualization, Writing – original draft, Writing – review & editing. HT: Data curation, Funding acquisition, Methodology, Project administration, Resources, Software, Supervision, Validation, Writing – original draft, Writing – review & editing.

## References

[1] Lagergren J Smyth E Cunningham D Lagergren P. Oesophageal cancer. Lancet (2017) 390(10110):2383–96. doi: 10.1016/S0140-6736(17)31462-9 28648400

[2] Kamangar F Shirkoohi R Shirkoohi R Haj-Mirzaian A Malekzadeh R Sepanlou SG. The global, regional, and national burden of oesophageal cancer and its attributable risk factors in 195 countries and territories, 1990-2017: a systematic analysis for the Global Burden of Disease Study 2017. Lancet Gastroenterol Hepatol (2020) 5(6):582–97. doi: 10.1016/S2468-1253(20)30007-8 PMC7232026 32246941

[3] Zhou M Wang H Zeng X Yin P Zhu J Chen W. Mortality, morbidity, and risk factors in China and its provinces, 1990-2017: a systematic analysis for the Global Burden of Disease Study 2017. Lancet (2019) 394(10204):1145–58. doi: 10.1016/S0140-6736(19)30427-1 PMC6891889 31248666

[4] van der Sluis PC Schizas D Liakakos T van Hillegersberg R. Minimally invasive esophagectomy. Dig Surg (2020) 37(2):93–100. doi: 10.1159/000497456 31096214

[5] Takahashi C Shridhar R Huston J Meredith K. Esophagectomy from then to now. J Gastrointest Oncol (2018) 9(5):903–9. doi: 10.21037/jgo.2018.08.15 PMC6219976 30505593

[6] Thomas PA. Milestones in the history of esophagectomy: from torek to minimally invasive approaches. Medicina (Kaunas) (2023) 59(10). doi: 10.3390/medicina59101786 PMC10608184 37893504

[7] Mallipeddi MK Onaitis MW. The contemporary role of minimally invasive esophagectomy in esophageal cancer. Curr Oncol Rep (2014) 16(3):374. doi: 10.1007/s11912-013-0374-9 24488547

[8] Biere SS van Berge Henegouwen MI Maas KW Bonavina L Rosman C Garcia JR. Minimally invasive versus open oesophagectomy for patients with oesophageal cancer: a multicentre, open-label, randomised controlled trial. Lancet (2012) 379(9829):1887–92. doi: 10.1016/S0140-6736(12)60516-9 22552194

[9] Straatman J van der Wielen N Cuesta MA Daams F Roig Garcia J Bonavina L. Minimally invasive versus open esophageal resection: three-year follow-up of the previously reported randomized controlled trial: the TIME trial. Ann Surg (2017) 266(2):232–6. doi: 10.1097/SLA.0000000000002171 28187044

[10] Shanmugasundaram R Hopkins R Neeman T Beenen E Fergusson J Gananadha S. Minimally invasive McKeown’s vs open oesophagectomy for cancer: A meta-analysis. Eur J Surg Oncol (2019) 45(6):941–9. doi: 10.1016/j.ejso.2018.11.017 30518481

[11] Watson TJ. Robotic esophagectomy: is it an advance and what is the future? Ann Thorac Surg (2008) 85(2):S757–9. doi: 10.1016/j.athoracsur.2007.11.046 18222211

[12] Boone J Schipper ME Moojen WA Borel Rinkes IH Cromheecke GJ van Hillegersberg R. Robot-assisted thoracoscopic oesophagectomy for cancer. Br J Surg (2009) 96(8):878–86. doi: 10.1002/bjs.6647 19591168

[13] Kim DJ Hyung WJ Lee CY Lee JG Haam SJ Park IK. Thoracoscopic esophagectomy for esophageal cancer: feasibility and safety of robotic assistance in the prone position. J Thorac Cardiovasc Surg (2010) 139(1):53–9.e1. doi: 10.1016/j.jtcvs.2009.05.030 19660280

[14] Puntambekar SP Rayate N Joshi S Agarwal G. Robotic transthoracic esophagectomy in the prone position: experience with 32 patients with esophageal cancer. J Thorac Cardiovasc Surg (2011) 142(5):1283–4. doi: 10.1016/j.jtcvs.2011.03.028 21530982

[15] van der Sluis PC Ruurda JP van der Horst S Goense L van Hillegersberg R. Learning curve for robot-assisted minimally invasive thoracoscopic esophagectomy: results from 312 cases. Ann Thorac Surg (2018) 106(1):264–71. doi: 10.1016/j.athoracsur.2018.01.038 29454718

[16] Manigrasso M Vertaldi S Marello A Antoniou SA Francis NK De Palma GD. Robotic esophagectomy. A systematic review with meta-analysis of clinical outcomes. J Pers Med (2021) 11(7). doi: 10.3390/jpm11070640 PMC8306060 34357107

[17] Bray F Ferlay J Soerjomataram I Siegel RL Torre LA Jemal A. Global cancer statistics 2018: GLOBOCAN estimates of incidence and mortality worldwide for 36 cancers in 185 countries. CA Cancer J Clin (2018) 68(6):394–424. doi: 10.3322/caac.21492 30207593

[18] He YT Li DJ Liang D Jin J Wen DG Chen WQ. [Estimated of esophageal cancer incidence and mortality in China, 2013]. Zhonghua Zhong Liu Za Zhi (2017) 39(4):315–20. doi: 10.3760/cma.j.issn.0253-3766.2017.04.016 28550676

[19] Torre LA Bray F Siegel RL Ferlay J Lortet-Tieulent J Jemal A. Global cancer statistics, 2012. CA Cancer J Clin (2015) 65(2):87–108. doi: 10.3322/caac.21262 25651787

[20] Chen MF Yang YH Lai CH Chen PC Chen WC. Outcome of patients with esophageal cancer: a nationwide analysis. Ann Surg Oncol (2013) 20(9):3023–30. doi: 10.1245/s10434-013-2935-4 23525703

[21] Chen W Zheng R Baade PD Zhang S Zeng H Bray F. Cancer statistics in China, 2015. CA Cancer J Clin (2016) 66(2):115–32. doi: 10.3322/caac.21338 26808342

[22] Luketich JD Pennathur A Awais O Levy RM Keeley S Shende M. Outcomes after minimally invasive esophagectomy: review of over 1000 patients. Ann Surg (2012) 256(1):95–103. doi: 10.1097/SLA.0b013e3182590603 22668811 PMC4103614

[23] Kato H Nakajima M. Treatments for esophageal cancer: a review. Gen Thorac Cardiovasc Surg (2013) 61(6):330–5. doi: 10.1007/s11748-013-0246-0 23568356

[24] Demarest CT Chang AC. The landmark series: multimodal therapy for esophageal cancer. Ann Surg Oncol (2021) 28(6):3375–82. doi: 10.1245/s10434-020-09565-5 33629251

[25] Wee JO Bravo-Iñiguez CE Jaklitsch MT. Early experience of robot-assisted esophagectomy with circular end-to-end stapled anastomosis. Ann Thorac Surg (2016) 102(1):253–9. doi: 10.1016/j.athoracsur.2016.02.050 27154153

[26] Grimminger PP Tagkalos E Hadzijusufovic E Corvinus F Babic B Lang H. Change from hybrid to fully minimally invasive and robotic esophagectomy is possible without compromises. Thorac Cardiovasc Surg (2019) 67(7):589–96. doi: 10.1055/s-0038-1670664 30216947

[27] Fujiwara H Shiozaki A Konishi H Otsuji E. Transmediastinal approach for esophageal cancer: A new trend toward radical surgery. Asian J Endosc Surg (2019) 12(1):30–6. doi: 10.1111/ases.12687 30681280

[28] Ericson J Lundell L Klevebro F Kamiya S Nilsson M Rouvelas I. Long-term weight development after esophagectomy for cancer-comparison between open Ivor-Lewis and minimally invasive surgical approaches. Dis Esophagus (2019) 32(4). doi: 10.1093/dote/doy075 30351390

[29] Wang Y Chen C. Survival following video-assisted thoracoscopic versus open esophagectomy for esophageal carcinoma. J Buon (2016) 21(2):427–33. 27273954

[30] van der Sluis PC Ruurda JP van der Horst S Verhage RJ Besselink MG Prins MJ. Robot-assisted minimally invasive thoraco-laparoscopic esophagectomy versus open transthoracic esophagectomy for resectable esophageal cancer, a randomized controlled trial (ROBOT trial). Trials (2012) 13:230. doi: 10.1186/1745-6215-13-230 23199187 PMC3564860

[31] Yip HC Shirakawa Y Cheng CY Huang CL Chiu PWY. Recent advances in minimally invasive esophagectomy for squamous esophageal cancer. Ann N Y Acad Sci (2020) 1482(1):113–20. doi: 10.1111/nyas.14461 32783237

[32] Osugi H Takemura M Higashino M Takada N Lee S Kinoshita H. A comparison of video-assisted thoracoscopic oesophagectomy and radical lymph node dissection for squamous cell cancer of the oesophagus with open operation. Br J Surg (2003) 90(1):108–13. doi: 10.1002/bjs.4022 12520585

[33] Kingma BF Grimminger PP van der Sluis PC van Det MJ Kouwenhoven EA Chao YK. Worldwide techniques and outcomes in robot-assisted minimally invasive esophagectomy (RAMIE): results from the multicenter international registry. Ann Surg (2022) 276(5):e386–e92. doi: 10.1097/SLA.0000000000004550 33177354

[34] Kingma BF de Maat MFG van der Horst S van der Sluis PC Ruurda JP van Hillegersberg R. Robot-assisted minimally invasive esophagectomy (RAMIE) improves perioperative outcomes: a review. J Thorac Dis (2019) 11(Suppl 5):S735–s42. doi: 10.21037/jtd.2018.11.104 PMC6503269 31080652

[35] Ozawa S Uchi Y Ando T Hayashi K Aoki T. Essential updates 2020/2021: Recent topics in surgery and perioperative therapy for esophageal cancer. Ann Gastroenterol Surg (2023) 7(3):346–57. doi: 10.1002/ags3.12657 PMC10154818 37152779

[36] Taurchini M Cuttitta A. Minimally invasive and robotic esophagectomy: state of the art. J Vis Surg (2017) 3:125. doi: 10.21037/jovs.2017.08.23 29078685 PMC5639027

[37] Gisbertz SS Hagens ERC Ruurda JP Schneider PM Tan LJ Domrachev SA. The evolution of surgical approach for esophageal cancer. Ann N Y Acad Sci (2018) 1434(1):149–55. doi: 10.1111/nyas.13957 30191569

[38] van der Sluis PC Tagkalos E Hadzijusufovic E Babic B Uzun E van Hillegersberg R. Robot-assisted minimally invasive esophagectomy with intrathoracic anastomosis (Ivor lewis): promising results in 100 consecutive patients (the european experience). J Gastrointest Surg (2021) 25(1):1–8. doi: 10.1007/s11605-019-04510-8 32072382 PMC7850999

[39] Chen J Liu Q Zhang X Yang H Tan Z Lin Y. Comparisons of short-term outcomes between robot-assisted and thoraco-laparoscopic esophagectomy with extended two-field lymph node dissection for resectable thoracic esophageal squamous cell carcinoma. J Thorac Dis (2019) 11(9):3874–80. doi: 10.21037/jtd.2019.09.05 PMC6790445 31656660

[40] Lux MM Marshall M Erturk E Joseph JV. Ergonomic evaluation and guidelines for use of the daVinci Robot system. J Endourol (2010) 24(3):371–5. doi: 10.1089/end.2009.0197 20073561

[41] Dunn D. Robotic-assisted surgery: A brief history to understand today’s practices. Aorn J (2022) 115(3):217–21. doi: 10.1002/aorn.13629 35213044

[42] Han J Davids J Ashrafian H Darzi A Elson DS Sodergren M. A systematic review of robotic surgery: From supervised paradigms to fully autonomous robotic approaches. Int J Med Robot (2022) 18(2):e2358. doi: 10.1002/rcs.2358 34953033

[43] Stringfield SB Parry LA Eisenstein SG Horgan SN Kane CJ Ramamoorthy SL. Experience with 10 years of a robotic surgery program at an Academic Medical Center. Surg Endosc (2022) 36(3):1950–60. doi: 10.1007/s00464-021-08478-y PMC8847263 33844089

[44] Singh TP Zaman J Cutler J. Robotic surgery: at the crossroads of a data explosion. World J Surg (2021) 45(12):3484–92. doi: 10.1007/s00268-021-06321-y 34635951

[45] Horgan S Berger RA Elli EF Espat NJ. Robotic-assisted minimally invasive transhiatal esophagectomy. Am Surg (2003) 69(7):624–6. doi: 10.1177/000313480306900716 12889629

[46] Silva JP Putnam LR Wu J Ding L Samakar K Abel S. Lower rates of unplanned conversion to open in robotic approach to esophagectomy for cancer. Am Surg (2022) 31348221104249. doi: 10.1177/00031348221104249 35611934

[47] Witek TD Brady JJ Sarkaria IS. Technique of robotic esophagectomy. J Thorac Dis (2021) 13(10):6195–204. doi: 10.21037/jtd.2020.02.43 PMC8575817 34795971

[48] Young A Alvarez Gallesio JM Sewell DB Carr R Molena D. Outcomes of robotic esophagectomy. J Thorac Dis (2021) 13(10):6163–8. doi: 10.21037/jtd-2019-rts-07 PMC8575850 34795967

[49] Xu Y Li XK Cong ZZ Zhou H Wu WJ Qiang Y. Long-term outcomes of robotic-assisted versus thoraco-laparoscopic McKeown esophagectomy for esophageal cancer: a propensity score-matched study. Dis Esophagus (2021) 34(9). doi: 10.1093/dote/doaa114 33150401

[50] Li B Yang Y Toker A Yu B Kang CH Abbas G. International consensus statement on robot-assisted minimally invasive esophagectomy (RAMIE). J Thorac Dis (2020) 12(12):7387–401. doi: 10.21037/jtd-20-1945 PMC7797844 33447428

[51] Fuchs HF Collins JW Babic B DuCoin C Meireles OR Grimminger PP. Robotic-assisted minimally invasive esophagectomy (RAMIE) for esophageal cancer training curriculum-a worldwide Delphi consensus study. Dis Esophagus (2022) 35(6). doi: 10.1093/dote/doab055 34382061

[52] van Hillegersberg R Boone J Draaisma WA Broeders IA Giezeman MJ Borel Rinkes IH. First experience with robot-assisted thoracoscopic esophagolymphadenectomy for esophageal cancer. Surg Endosc (2006) 20(9):1435–9. doi: 10.1007/s00464-005-0674-8 16703427

[53] Suda K Ishida Y Kawamura Y Inaba K Kanaya S Teramukai S. Robot-assisted thoracoscopic lymphadenectomy along the left recurrent laryngeal nerve for esophageal squamous cell carcinoma in the prone position: technical report and short-term outcomes. World J Surg (2012) 36(7):1608–16. doi: 10.1007/s00268-012-1538-8 22392356

[54] Cerfolio RJ Bryant AS Hawn MT. Technical aspects and early results of robotic esophagectomy with chest anastomosis. J Thorac Cardiovasc Surg (2013) 145(1):90–6. doi: 10.1016/j.jtcvs.2012.04.022 22910197

[55] Osaka Y Tachibana S Ota Y Suda T Makuuti Y Watanabe T. Usefulness of robot-assisted thoracoscopic esophagectomy. Gen Thorac Cardiovasc Surg (2018) 66(4):225–31. doi: 10.1007/s11748-018-0897-y 29397486

